# Host–Multi-Pathogen Warfare: Pathogen Interactions in Co-infected Plants

**DOI:** 10.3389/fpls.2017.01806

**Published:** 2017-10-25

**Authors:** Araz S. Abdullah, Caroline S. Moffat, Francisco J. Lopez-Ruiz, Mark R. Gibberd, John Hamblin, Ayalsew Zerihun

**Affiliations:** ^1^Centre for Crop and Disease Management, Department of Environment and Agriculture, Curtin University, Bentley, WA, Australia; ^2^Institute of Agriculture, University of Western Australia, Perth, WA, Australia

**Keywords:** multiple infections, plant defense to co-infection, pathogen competition, pathogen cooperation, pathogen coexistence, niche heterogeneity

## Abstract

Studies of plant–pathogen interactions have historically focused on simple models of infection involving single host-single disease systems. However, plant infections often involve multiple species and/or genotypes and exhibit complexities not captured in single host-single disease systems. Here, we review recent insights into co-infection systems focusing on the dynamics of host-multi-pathogen interactions and the implications for host susceptibility/resistance. In co-infection systems, pathogen interactions include: (i) Competition, in which competing pathogens develop physical barriers or utilize toxins to exclude competitors from resource-dense niches; (ii) Cooperation, whereby pathogens beneficially interact, by providing mutual biochemical signals essential for pathogenesis, or through functional complementation via the exchange of resources necessary for survival; (iii) Coexistence, whereby pathogens can stably coexist through niche specialization. Furthermore, hosts are also able to, actively or passively, modulate niche competition through defense responses that target at least one pathogen. Typically, however, virulent pathogens subvert host defenses to facilitate infection, and responses elicited by one pathogen may be modified in the presence of another pathogen. Evidence also exists, albeit rare, of pathogens incorporating foreign genes that broaden niche adaptation and improve virulence. Throughout this review, we draw upon examples of co-infection systems from a range of pathogen types and identify outstanding questions for future innovation in disease control strategies.

## Introduction

Plant pathology has focused predominantly on single host-single disease interactions. Whilst this simplification has proved useful, plants in nature interact with multiple pathogen species/genotypes (Kozanitas et al., 2017; Tollenaere et al., 2017). This complex interaction, known as co-infection, is of particular interest since it tends to alter the course of the disease and the severity of expression (i.e., overall virulence) and has been the subject of a recent review in plant epidemiology (Tollenaere et al., 2016).

Three key interactions can cause damage in co-infected plants: host–pathogen, pathogen–pathogen, and host-multiple-pathogen complexes. Host–pathogen interactions are well-studied and are generally detrimental to the plant resulting in reduced fitness (Brown, 2015). In contrast, pathogen–pathogen and host-multiple-pathogen interactions are less studied. These interactions can lead to various results: antagonism, synergism, coexistence, mutualism, or cooperation (**Table 1**). The level of disease damage the plant experiences varies depending on the outcome of the interactions and the corresponding host responses. For example, several strains of *Pseudomonas* bacteria secrete antimicrobial compounds that are antagonistic to sensitive pathogens within the host (Walsh et al., 2001). Many such compounds are also phytotoxic and may exacerbate the level of disease damage (Maurhofer et al., 1992). Furthermore, some pathogens, such as the biotroph *Blumeria graminis* f. sp. *tritici* and the necrotroph *Zymoseptoria tritici* of wheat, do not interact directly to cause damage to the host as one pathogen can inhibit the development of the other (Orton and Brown, 2016). This inhibition can be so profound that the plant plays an active role in promoting the growth of disease suppressive pathogens (Smith et al., 1999). Therefore, moving beyond how heterogeneity of infection influences overall virulence requires a holistic understanding of how a host responds to co-infection and how pathogens interact and coexist.

**Table 1:** Examples of pathogen–pathogen interactions in various plants and pathogen species.

Pathogen species	Host	Co-infection type	Interaction type	Reference
*Pseudomonas syringae*/*Alternaria brassicicola*	Arabidopsis	Synchronous	Synergistic	Spoel et al., 2007
*Fusarium oxysporum*/*Pseudomonas fluorescens*	Wheat	Synchronous	Synergistic	Notz et al., 2002
*Fusarium oxysporum*^(Fo47)^/*Fusarium oxysporum*^(Fol8)^	Tomato	Asynchronous	Antagonistic	Aime et al., 2013
*Pseudomonas putida*/*Botrytis cinerea*	Field bean	Synchronous	Antagonistic	Ongena et al., 2005
*Leptosphaeria maculans*/*Leptosphaeria biglobosa*	Oilseed rape	Asynchronous	Coexistence	Toscano-Underwood et al., 2003
*Zymoseptoria tritici*/*Blumeria graminis tritici*	Wheat	Synchronous/asynchronous	Antagonistic	Orton and Brown, 2016
*Fusarium verticillioides*/*Ustilago maydis*	Maize	Synchronous	Antagonistic	Jonkers et al., 2012
*Fusarium graminearum*/*Phoma* sp.	Finger millet	Synchronous	Antagonistic	Mousa et al., 2015
*Fusarium oxysporum*/*Pseudomonas fluorescens*	Tomato	Synchronous	Synergistic	Kamilova et al., 2008
*Rhizopus microspores*/*Burkholderia* sp.	Rice	Synchronous	Symbiosis	Partida-Martinez and Hertweck, 2005
Rice yellow mottle virus/*Xanthomonas oryzae*	Rice	Synchronous/asynchronous	Synergistic	Tollenaere et al., 2017

Recent advances in genomic and molecular techniques have led to new insights into host–pathogen dynamics. For example, metagenomics and microbial tag sequencing have created novel opportunities for studying the wide range of pathogens associated with a single host (Petrosino et al., 2009; Tollenaere et al., 2012). These tools have provided insights into the prevalence of multiple infections in the field, and current knowledge indicates that the extent of co-infection can be significant in some pathosystems (Susi et al., 2015; Tollenaere et al., 2017). Progress in this area has revealed that co-infections can lead to several outcomes: (i) competitive exclusion where over time one pathogen excludes the other (Al-Naimi et al., 2005); (ii) mutualistic coexistence in which both pathogens receive benefits from the interaction (Mordecai et al., 2016); and (iii) emergence of new recombinant genomes where one pathogen incorporates a complementary gene set from another pathogen, leading to large-scale epidemics (Friesen et al., 2006). Indeed, populations of one pathogen may modify host environments to the advantage/disadvantage of other pathogens, affecting their frequencies and persistence within a pathogenic population (Perefarres et al., 2014). Hence, understanding within host–pathogen interaction is crucial for the prediction of long-term dynamics of multiple disease outcomes.

In this review, we discuss recent insights into within-host disease diversity and dynamics of pathogen interactions focusing on the current understanding of pathogen competition and cooperation, and the mechanisms that allow long-term coexistence to occur. We draw upon examples of co-infections from a range of pathogen types that provide useful insights for understanding the evolution of pathogen interactions and coexistence.

## Plant Defense Responses to Co-Infection

The overwhelming majority of studies on plant defense responses to pathogenic infections have been performed on single plant-disease pathosystems. However, under conducive conditions, plants frequently encounter multiple pathogens, often with various modes of action. Hence, a successful plant defense system will incorporate several resistance (*R*) genes that coordinate a response to multiple attacks. The genomes of plants encode a coordinated array of *R*-genes that permit recognition of the pathogen and a rapid defense response (Dangl and Jones, 2001). Prioritization of defense may occur, leading to larger investments in defense metabolites against certain pathogens depending on their modes of action (Hacquard et al., 2016; Castrillo et al., 2017). This raises the question: does an infection by one-pathogen influence a host defense to subsequent infection by other pathogens?

### Pathogen-Triggered Host Susceptibility

Some pathogenic infections can be detrimental to the defense systems predisposing the plant to subsequent secondary infections. For example, infection of Arabidopsis by the foliar bacterium *Pseudomonas syringae* renders plants vulnerable to invasion by the necrotrophic ascomycete *Alternaria brassicicola* (Spoel et al., 2007). Infection by the biotrophic oomycete *Albugo candida* suppresses Arabidopsis defenses, permitting subsequent infections by several otherwise avirulent pathogens (Cooper et al., 2008). Similarly, infection of maize by the phytopathogenic fungi *Fusarium verticillioides* facilitates infection by several related fungi, through the suppression of production of major secondary defense metabolites in the plant host (Saunders and Kohn, 2008). The mechanisms that lead to the suppression of defense have been defined in some cases. For example, the natriuretic peptide receptor NPA expressed by *P. syringae* permits subsequent infection by virulent *A. brassicicola* in Arabidopsis through downregulating a large range of defense-related genes (Spoel et al., 2003; Cooper et al., 2008). Similarly, fusaric acid secreted by *F. oxysporum* suppresses expression of genes that regulate the antimicrobial activity of 2,4-diacetylphloroglucinol and predisposes wheat to *P. fluorescens* infection (Notz et al., 2002).

### Pathogen-Triggered Host Immunity

Some pathogenic infections can enhance the defensive capacity of their hosts and activate responses against subsequent attacks. For example, infection by the foliar bacterium *P. fluorescens* suppresses flagellin-triggered defense in Arabidopsis via apoplastic secretion of low-molecular-weight defense metabolites (Millet et al., 2010). Upon exposure to the bacterium, the defense system of the plant is locally suppressed; though a defense-signaling cascade develops systematically and spreads across infected plant parts conferring resistance to subsequent attacks (Van Der Ent et al., 2009). Some root infections can confer resistance by forming rhizosphere networks that connect infected plants and signal-induced resistance to neighboring plants (Song et al., 2010). Similarly, induced resistance has been reported for co-infections by species of the same genus, where non-pathogenic *F. oxysporum* primed tomato plants to pathogenic *F. oxysporum* in a vaccine-like fashion (Aime et al., 2013). The molecular mechanisms involved in this priming have not been fully elucidated, although direct antagonism, detoxification of pathogen effectors and elevated expressions of plant defense-related genes have been recorded (Aime et al., 2013; Ravensdale et al., 2014; Conrath et al., 2015).

### Crosstalk among Jasmonate, Ethylene, and Salicylate

Recently, there has been growing interest in plant defense responses to co-infection at the hormonal level. This involves a comparative pathway analysis following infections by multiple organisms. Expression levels of genes responsive to jasmonic acid (JA), ethylene (Et), and salicylic acid (SA) are commonly measured in such analyses. Generally, JA and Et are considered to be mutual pathways linked to defense against necrotrophic pathogens such as *Botrytis cinerea* (Grant and Jones, 2009). SA, on the other hand, is often linked to defense against biotrophic pathogens such as *P. syringae* (Glazebrook, 2005). Antagonistic crosstalk between JA/ET and SA is well-documented (Pieterse et al., 2009; Robert-Seilaniantz et al., 2011), permitting the plant to mount the appropriate defense responses to the attacking pathogen. In Arabidopsis, elevated expression of JA/Et-responsive genes resulted in antagonistic effects on SA-responsive genes and increased plant resistance to *B. cinerea* (Moffat et al., 2012). Similarly, exogenously applied SA revealed antagonist effects on expressions of JA-responsive genes but simultaneously increased Arabidopsis resistance to *P. syringae* (Gazzarrini and Mccourt, 2003). Pathogens have evolved sophisticated mechanisms to exploit this antagonism and counter host defense responses. For example, the polyketide phytotoxin coronatine secreted by *P. syringae* is structurally analogous to jasmonoyl-isoleucine and can bind to JA receptors, hijacking the JA mediated defense and causing disease susceptibility to *P. syringae* (Katsir et al., 2008). Similarly, the avirulent effector AvrPtoB produced by *P. syringae* disrupts hormonal signaling components in Arabidopsis creating the potential for vulnerability to subsequent infections (De Torres-Zabala et al., 2007).

Although SA and JA signaling can be activated separately, recent studies have shown varying degrees of involvement of both pathways depending on plant–pathogen combinations. An elegant comparative transcriptomics study revealed significant overlap in Arabidopsis responses to a set of biotrophic and necrotrophic attackers (De Vos et al., 2005). Global gene expression analyses revealed that all Arabidopsis-attackers stimulated JA biosynthesis (De Vos et al., 2005). Similarly, infection by the non-necrotroph *P. syringae* induced a JA-mediated defense in Arabidopsis localized to infected regions (Cui et al., 2005). These results suggest the model of SA-mediated defense against biotrophs, and JA/Et-mediated defense against necrotrophs is too simplistic. The defense responses are likely to be fine-tuned to particular plant–pathogen combinations. There is much yet to be learned about mechanisms that allow for these differences, and this is an active area of research.

## Microbial Lifestyle and Plant Defense

Many plant microbes can have latent pathogenic capacities and specialize in causing disease when host conditions are suitable. These microbes can be commensal, living benignly with limited or no damage to the plant, but may become pathogenic should host physiological conditions change (Hentschel et al., 2000). The foliar fungi *Ramularia collo-cygni* is a good example of a microbe that may be either commensal or pathogenic depending on the developmental stage of its barley host. The fungus becomes more aggressive during the late stages of barley development, and physiological events associated with flowering are thought to trigger the shift to necrotrophic lifestyle (Mcgrann et al., 2016). Even closely related members of a genus, notably species belonging to *Colletotrichum*, can be symbiotic under certain conditions and highly pathogenic under other conditions (Hiruma et al., 2016). Similarly, the switch to the pathogenic lifestyles for many microbes only occurs in defense-compromised plants following other aggressive disease encounters (Lahrmann et al., 2015; Fesel and Zuccaro, 2016). For instance, infection by highly virulent *P. syringae* induces systematic susceptibility in Arabidopsis stimulating the pathogenic capacity of an otherwise avirulent *P. syringae* isolate (Thomma et al., 2001).

The defense systems of the plant are as likely to recognize and respond to commensal microbes as they are to pathogenic ones (Dangl and Jones, 2001). Pathogenic microbes can suppress the defense systems of the plant to the extent that disease develops. Whilst commensal microbes can also suppress the defense systems of the plant; this is not usually to the extent that severe disease develops. Therefore, pathogenic interactions are those that outweigh the defense systems of the host resulting in disease, whereas commensal interactions are balanced and remain asymptomatic (reviewed in Schulz and Boyle, 2005). Whether a particular plant–microbe interaction leads to a disease depends on host factors that maintain the balance between host defense and pathogen virulence (Schulz et al., 1999). If this balance shifts to favor the microbe, commensal interactions can then become pathogenic. For example, suboptimal nutrition has been shown to weaken the defense systems of plants resulting in disease and growth reduction caused by an otherwise avirulent microbe (Kuldau and Yates, 2000). Similarly, highly pathogenic encounters with a necrotrophic *P. syringae* compromised the defense balance in Arabidopsis and rendered the host vulnerable to infection by avirulent *P. syringae* (Thomma et al., 2001). Hence, plant defense responses to microbes are highly unpredictable, and yet undiscovered mechanisms may determine the outcome of such interactions.

What factors the altered microbial metabolism between commensal and pathogenic is a promising area for future research. Key factors for such shifts are not well-studied; although, sudden changes in temperature and/or humidity are possible triggers (Mcgrann et al., 2016). Some changes in metabolism can be induced by pathogen signals that alter host defense responses. *Moniliophthora perniciosa* is a fungal species often found in cocoa shoots as an endophyte living benignly without causing apparent disease. Colonization of cocoa shoots by this fungus gradually increases SA production while decreasing JA production triggering a lifestyle shift and rendering the plant susceptible to the necrotrophic phase of the fungus (Chaves and Gianfagna, 2006). *M. perniciosa* is thought to have acquired the ability to produce SA to facilitate active competition with other plant microbes and initiate the transition to more aggressive lifestyles (Bezemer et al., 2006). Regardless of what the actual triggers for this shift are, it may indicate a decline in host available resources that are vital for pathogen survival and reproduction creating a potential for multi-pathogen competition. It remains unclear how plants discriminate and respond appropriately to closely related microbes with variable metabolism.

## Multi-Pathogen Competition

Recent advances in metagenomics have highlighted the vast diversity of the community in which plant pathogens reside (Schenk et al., 2012). Due to the complex nature of these communities and the limited host resources, pathogens often enter into fierce competition. Fundamentally, competition between coexisting pathogens occurs for growth- and fitness-limiting resources. Resource competition involves utilization of limited host nutrients by one pathogen that then restricts supply to other pathogens sharing the same host. Monod (1949) was the first to point out the relationship between nutrients and pathogen growth: within a defined space in which all nutrients are provided, pathogens may stably coexist. Suboptimal nutrition leads to competition whereby some species may dominate. The severity and type of competition is determined by the consumption of nutrients over time, and this principle has been applied to plant and animal populations as an explanation of the dynamics of competing individuals (Adee et al., 1990; Chesson, 2000).

**FIGURE 1:**
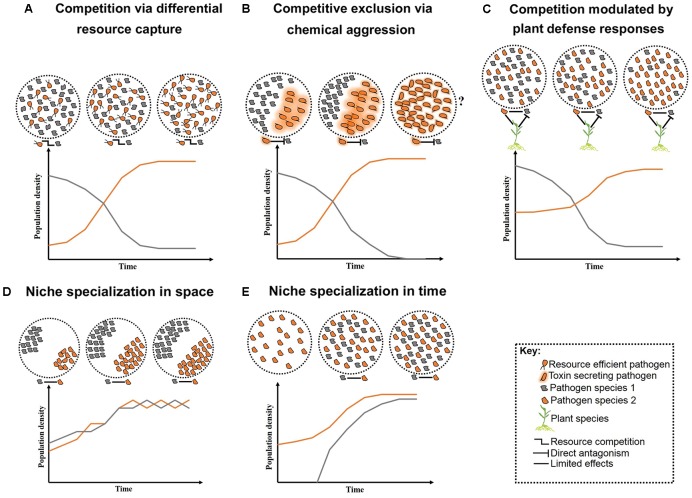
Schematic representation of long-term pathogen–pathogen interactions. Competition over growth limiting resources and/or host niches can initially create high diversity, but low stability. Overtime, competition restricts diversity and allows competitive species to outgrow less competitive ones **(A–C)**. Alternatively, neutral interactions occur when pathogens co-reside distinct niches within their host **(D)** or arrive at various time intervals **(E)**. Question marks represent less studied cases where toxin production may lead to reduce fitness in the absent of competitors.

In nutritionally defined niches, such as plant leaves, pathogens with similar nutritional requirements compete for finite resources (West et al., 2006). Competition under such conditions may lead to selection for the more virulent species, or conversely, all associated pathogens may suffer (Rankin et al., 2007). For example, when maize was co-infected by *Ustilago maydis* and *F. verticillioides*, initially both fungal species had increased growth followed by decreased growth over time due to the depletion of nutrient resources (Jonkers et al., 2012). Pathogen traits such as cell-wall adhesion can support greater nutrient acquisition and provide competitive advantage (Hibbing et al., 2010). Adherent cell walls can enable greater resource capture efficiency even when growth substrates are present at low concentrations (**Figure 1A**). Similarly, pathogens that can halt certain metabolic processes, such as toxin production, when the necessary nutrients are exhausted, may show a greater competitive advantage (Glenn et al., 2008). Improved nutrient acquisition can also be achieved by the release of cell-wall degrading enzymes or toxins to sequester host nutrients (Greenberg and Yao, 2004; Rohmer et al., 2011). However, these compounds may also provide a competitive advantage to other opportunistic pathogens that do not have to bear the energetic costs for their production (Hentzer et al., 2003; Cornelis and Dingemans, 2013; Ghoul et al., 2014).

More aggressive forms of competition between pathogens include direct chemical exclusion (**Figure 1B**). A classic example of chemical aggression includes tenuazonic acid secreted by the finger millet colonizing endophyte *Phoma* sp. which prevents growth of several pathogens including the toxigenic fungus *F. graminearum* (Mousa et al., 2015, 2016a). Mechanical aggression also occurs through disruption of cell membranes and formation of multilayer physical barriers limiting growth and infection success of competitors. For example, the root-inhabiting bacteria *Enterobacter* sp. forms specialized root-pathogen structures to prevent infection by *F. graminearum* in finger millet (Mousa et al., 2016b). A molecular mechanism involved in pathogen competition has been identified. The N-alkylated benzylamine secreted by the non-pathogenic bacterium *P. putida* acts as a lytic enzyme that inhibits fungal growth of *B. cinerea* in field bean (Ongena et al., 2005). Competition can also occur indirectly, mediated by the host through targeted defense responses against at least one pathogen (**Figure 1C**). However, the targeted defense can also provide a competitive advantage to pathogens that can counteract the recognition process of the host or that are capable of evading the plant defense system (Ren and Gijzen, 2016).

An expected outcome of competition is a localized reduction in diversity and concurrent specialization of pathogens to various tissues and/or host species – i.e., niche specialization. An individual plant may contain several niches in which pathogens can exist (**Figure 1D**), and heterogeneity in the biology and epidemiology of pathogens may permit niche specialization (**Figure 1E**). For example, differences in disease onset resulted in temporal separation and stable coexistence between two related fungal pathogens of canola, *Leptosphaeria maculans* and *L. biglobosa* (Toscano-Underwood et al., 2003). Niche specialization can reduce the severity of competition between pathogens permitting coexistence (Fitt et al., 2006), although pathogens occupying various niches within a plant may interact indirectly by stimulating a common host defense response (Aghnoum and Niks, 2012). Nevertheless, on an evolutionary timescale, competition may result in exclusion, enabling species to coexist when arriving at various times (Fitt et al., 2006; Pfennig and Pfennig, 2009; Perefarres et al., 2014). However, when displacement leads to the exclusion of the less competitive pathogen, the newly evolved highly competitive species may compete with other pathogens for their optimal niches (Perefarres et al., 2014). This situation may arise if a species integrates foreign genes allowing the invasion of novel niches (Friesen et al., 2006). Individuals of other species would then have fewer unoccupied niches in which to gain footholds, resulting in potentially large scale epidemics (Perefarres et al., 2014). Although the role of the integration of foreign genes in niche specialization and expansion is less clear, interspecies acquisition of the *ToxA* gene has allowed *Pyrenophora tritici*-*repentis* to infect ToxA-sensitive wheat varieties (Friesen et al., 2006).

## Opportunistic Resource Exploitation

Virulence of many plant pathogens depends on secretion of growth promoting factors that subvert host defenses and improve their own nutrient uptake. These growth-promoting factors are often deployed into the extracellular matrix of the host and may hence be indirectly accessible by other pathogens in a local community (Harrison et al., 2006). In such cases, producing species can be vulnerable to exploitation by opportunists that can utilize virulence factors of their neighbors without contributing to the production of these factors. *P. syringae* provides a good example where common virulence factors give rise to opportunistic exploitation. Virulence in this pathogen is facilitated by the type III secretion system (T3SS), a needle-like apparatus that enables injection of toxins into host leaf mesophyll (Hauck et al., 2003). Virulence factors required for host manipulation by this pathogen are expressed in a bistable fashion, leading to a slow-growing toxin-secreting (T3SS+) strain and a fast-growing toxin-lacking (T3SS-) strain (Barrett et al., 2011). Co-infection experiments initiated with wild-type Arabidopsis revealed a growth advantage to the less virulent T3SS- strain resulting from the opportunistic exploitation of the toxins secreted by T3SS+ (Barrett et al., 2011). Iron-scavenging siderophores provide an additional example whereby co-infection gives rise to opportunistic exploitation. Many opportunistic bacteria can take up heterologous siderophores, diverting iron away from siderophore producing strains and simultaneously passing the cost of production to co-infecting neighbors (Khan et al., 2006). Hence, siderophore-producing bacteria are vulnerable to opportunistic bacteria that are able to utilize heterologous iron-binding products (Khan et al., 2006). The bacteria are not subject to the energetic costs associated with siderophore production and can outgrow more virulent producing genotypes (Alizon and Lion, 2011). Indeed, during co-infection of Arabidopsis with several strains of *P. aeruginosa*, siderophore lacking bacteria evolved more rapidly and dominated siderophore producers in iron-limited conditions (Khan et al., 2006).

## Multi-Pathogen Cooperation

**FIGURE 2:**
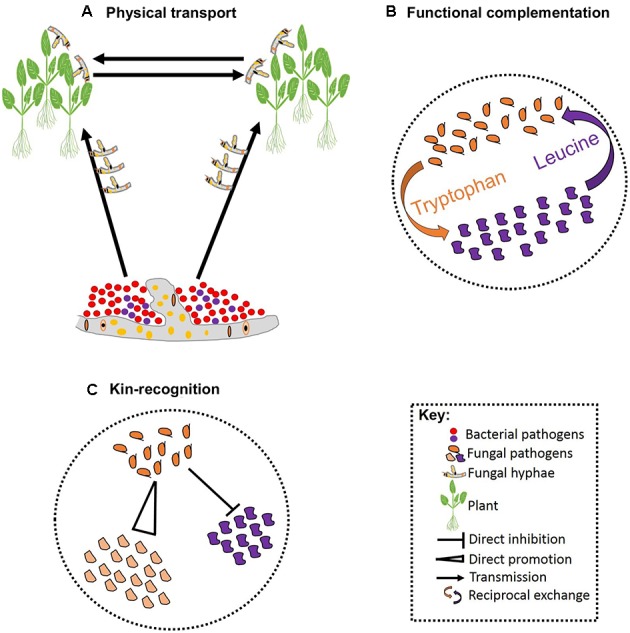
Schematic representation of pathogen–pathogen cooperation. Some bacteria can establish symbiotic associations with fungi that allowing them to exploit new niches and move across infected hosts **(A)**. Cooperation can be vital for survival of both parties involved leading to reciprocal exchange of vital growth substrates **(B)**. Cooperation can also involve kinship and some pathogens can facilitate fitness of their kin while restricting fitness of non-kin **(C)**.

Besides competition, pathogens may also cooperate in associations that are essential for pathogenesis. These associations can be facilitated by biophysical and/or biochemical means. For example, hyphae of the fungal ascomycete *Didymella bryoniae* physically transported four bacterial species that co-infect Styrian oil pumpkin (Grube et al., 2011; **Figure 2A**). Samples including both the fungus and bacteria have been recovered from the field, indicating that mutualistic effects on pathogenesis may occur in nature (Grube et al., 2011). The association between *Rhizopus microsporus* and *Burkholderia* sp. is an example where a precise biochemical fungal-bacterial association has been identified. *R. microsporus* is a highly destructive zygomycetous fungus that causes blight in rice seedlings. The fungus is thought to secrete a phytotoxin known as rhizoxin; although no standard polyketide synthesis genes could be identified in the fungus genome (Partida-Martinez and Hertweck, 2005). The rhizoxin was, however, found to be secreted by the endosymbiotic bacteria that are harbored by the fungus. Thus, it appears that *R. microsporus* owes its pathogenicity to the presence of an endosymbiont bacteria belonging to the genus *Burkholderia* (Partida-Martinez and Hertweck, 2005). Intimate biochemical fungal-bacterial symbiosis occurs when both a recognition system and a molecular dialog bind the two. For instance, recognition of fusaric acid secreted by certain isolates of the fungus *F. oxysporum* stimulates growth in the bacterial pathogen *P. fluorescens* in tomato (De Weert et al., 2004; Kamilova et al., 2008).

Endophytes, although less studied in the context of co-infection, has been considered as an additional component to the multi-pathogen symbiosis (Aime et al., 2013). Endophytes colonize internal plant tissues without causing visible disease and produce antimicrobial compounds that enhance general plant fitness and affect pathogen interactions directly and indirectly (for review, see Partida-Martínez and Heil, 2011). Among the most studied endophytes is the biocontrol agent *F. oxysporum*. This root-inhabiting fungus produces antimicrobial compounds that enhance plant resistance to pathogenic *F. oxysporum* (Aime et al., 2013). Recent microscopic and molecular investigations revealed the presence of a consortium of ectosymbiotic bacteria associated with *F. oxysporum* and crucial for the biocontrol properties of the fungus (Minerdi et al., 2008). However, when the fungus was cured of its associated bacteria, the biocontrol strain became pathogenic (Minerdi et al., 2008). This demonstrates that mutualistic cooperation that involves endophytes is not clear-cut, and complex dynamics involving other microbes may alter the properties of endophytic interactions.

Regardless, mutualistic cooperation between pathogens can have major epidemiological implications, and certain plant pathogens are only host destructive when they cooperate with other independent pathogens. For example, obligate mutualism between maize dwarf mosaic virus and wheat streak mosaic virus causes lethal maize necrosis – neither of these pathogens is known to cause lethal necrosis alone (Uyemoto, 1983). Similarly, co-infection of tobacco mosaic virus and potato virus cause defoliation streak and a high rate of mortality in young tomato leaves (Hull, 2014). Cooperation can also enhance pathogen persistence by supporting greater reproduction rates, increasing the chance of the host being a source of inoculum in the next season (Fondong et al., 2000). Co-infection of *Nicotiana benthamiana* plants with two strains of the cassava mosaic virus showed symptoms covering all leaves, while single-strain infection exhibited partial coverage and some leaves remained asymptomatic (Fondong et al., 2000).

Reciprocal exchange of growth substrates is a common strategy through which pathogens establish stable cooperation. Such cooperative associations are prominent when resources are deficient, and one species produces the growth substrates required by the other (**Figure 2B**). Hence, cooperation in such cases is vital for the success of all species involved (Hoek et al., 2016). In addition, there are a few well-characterized studies of altruistic cooperation between closely related species. Altruistic cooperation occurs when co-infection leads to the reduced availability of resources per species, such that the less competitive species experience reduced reproduction rates preserving resources for the reproduction of the other species (West et al., 2002; Kummerli et al., 2009). Hence, the reproductive advantage of the competitive species is dependent on the other species (**Figure 2C**). Hamilton’s kin-selection theory provides a plausible explanation for the altruistic association between relatives: by helping a close relative, a pathogen is indirectly passing its genes to the next generation (Hamilton, 1963). This can occur when the degree of relatedness between the benefactor and the beneficiary is high, and the benefit outweighs the cost of the cooperation (Eberhard, 1975; Chao et al., 2000). Coexisting pathogens that exhibit altruism may display rapid evolution to overcome pesticides as a result of the reproductive advantage of the more competitive species. Furthermore, initiating defense responses against various pathogens acting together may prove costly to host resources and defense systems, although evidence for such interactions in plant pathogens is currently lacking.

## Evolution of Pathogens in Co-Infections

Recent research into pathogen interactions has considered the genetic adaptation that close proximity facilitates. It has been suggested that genomes of some species display reduced mutation rates as an adaptation strategy to the presence of other coexisting species (Cordero and Polz, 2014). Fungi isolated from tree-hole rainwater pools exhibited similar resource acquisition strategies that should lead to severe competition. However, culturing these isolates on media that resembled their natural habitat resulted in a reciprocal exchange of growth substrates (Madsen et al., 2016). When multiple species are in close proximity, genetic recombination may occur through the fusion of haploid cells. For instance, reshuffling of alleles between genetically distant fungi resulted in novel genetic diversity in *Z. pseudotritici* (Stukenbrock et al., 2012). Novel hybrids often display new characteristics that enable colonization of previously unexploited niches. For example, the horizontal transfer of the host-specific gene *ToxA* from *P. nodorum* to *P. tritici-repentis* allows *P. tritici-repentis* to invade ToxA sensitive wheat cultivars on a large scale (Friesen et al., 2006).

Co-infection can also provide a selective advantage for the adaptation of low-frequency pathogen populations. For example, the persistence of non-virulent strains of *P. syringae* was partially linked to their growth advantage through coexistence with virulent strains (Barrett et al., 2011). Similarly, laboratory-based co-infection studies demonstrated a fitness advantage to less competent pathogens conditional on their coexistence with fully-adapted pathogens (Bashey, 2015). Perhaps the simplest explanation for such fitness advantage would be a mass-action mechanism – i.e., more disease load within the plant implies greater disease transmission rates and greater infection opportunities for both species (Perefarres et al., 2014). A less direct explanation, especially applicable for viruses, may involve heteroencapsidation. This may occur when two co-infecting species display sufficient genetic variability in which case the more competent species may complement the less competent species providing benefits in accumulation and transmission within and among plants (Perefarres et al., 2014). Direct testing of mechanisms by which co-infection contributes to the maintenance of pathogen diversity is an exciting research area.

## Evolution of Virulence in Co-Infecting Pathogens

Current knowledge suggests that virulence is an inevitable requirement for host exploitation. Evolution of virulence can be constrained by the reproduction rate of a pathogen (Alizon et al., 2013). Hence, increased virulence may initially be advantageous, but subsequent consequences resulting from increased host mortality are not (Chao et al., 2000; Ebert and Bull, 2003). This is particularly true in pathosystems where both the host and pathogen have comparable generation times, in which case the costs of increased virulence are spread among all species sharing the host – the so-called tragedy of the commons (Hardin, 1968). Under co-infection conditions, pathogens are thought to utilize host-limited resources more efficiently with natural selection favoring the coexistence of pathogens that are less harmful to their hosts (Van Baalen and Sabelis, 1995; Brown et al., 2002). Early insights into the evolution of virulence were provided by the classical three-way model of Levin and Pimentel (1981) which included a host and two pathogens differing in virulence. Based on this model, when two pathogens invade the same host, virulence of one species is always considered relative to the virulence of the other species (Levin and Pimentel, 1981; Brown et al., 2002).

Early during co-infection, the more virulent pathogen may quickly take over. Nevertheless, both more and less virulent pathogens can coexist (Van Baalen and Sabelis, 1995). *In vitro* experiments suggest that coexistence between two pathogen species varying in virulence can occur. In one system, the population of the more virulent pathogen *U. maydis* was able to reach higher frequencies over the less virulent species *F. verticillioides*, though both species coexisted stably (Jonkers et al., 2012). However, when the same system was conducted *in planta* using maize, the less virulent species experienced lower resistance from the host and hence gained a competitive advantage over the more virulent pathogen (Estrada et al., 2012). As the less virulent species dominate, local competition of some degree takes place, and the balance may shift to favor the more virulent species (Macho et al., 2007). Once again, as the more virulent pathogen becomes abundant, plant defense systems prioritize responses against the more virulent pathogen, thereby indirectly allowing the less virulent pathogen to regain lost ground (De Roode et al., 2005; Thieme, 2007; Cobey and Lipsitch, 2013). This cycle is key for pathogen coexistence in space and time and implies, from an evolutionary perspective, that neither too high nor too low levels of virulence are advantageous.

## Concluding Remarks

If we are to make significant progress in plant disease management, research efforts should embrace field representative systems including multiple-pathogen infections. The role of pathogen–pathogen interactions and their impact on plant defense systems should increasingly be recognized as a priority of equal importance to studying single plant–pathogen interactions. Although rare, interaction between pathogens potentially allows the exchange of genes encoding virulence factors broadening pathogen infection strategies and allowing them to exploit new niches (Friesen et al., 2006). However, some interactions can alert the defense system of the plant making subsequent infections less likely. Progress in utilizing pathogen–pathogen interactions for developing holistic disease management strategies has been underwhelming, indicating an area that requires attention (Martinez-Medina et al., 2016).

In co-infection, pathogens can produce antimicrobial compounds toxic to other pathogens sharing the same host. However, whether production of such compounds truly represents an adaptation strategy to competition is unclear. It would be debilitating for a pathogen to produce compounds toward future threats if these threats are not realized. Such pathogens would be selected against under a reasonable assumption that the pathogen must target direct enemies or risk wasting vital resources. Nevertheless, pathogens can coexist and share a host, mainly due to conditions favoring the occurrence of multiple pathogens. An outstanding question is how changes in natural (e.g., climatic) and man-made (e.g., new varieties with polygenic or major gene resistances) conditions alter coexistence on the long-term. Changes to conditions may favor one pathogen over another, leading to potential invasion by large-scale aggressive pathogens. Other challenges that require future attention is cases of below- and above-ground co-infections. Pathogens colonizing various plant parts can interact via systemic host defenses, making studying these interactions particularly intriguing (Filgueiras et al., 2016). Other challenging questions that may have significant epidemiological implications are cases where pathogens invade novel niches/species. The metabolic changes that are required for a pathogen to optimize nutrient acquisition from novel niches remain unclear. High-throughput multi-species transcriptomics and metabolomics should help to unravel some of the mechanisms. Such methods will provide a better understanding of pathogen interactions allowing the development of disease control measures against multiple pathogens.

## Author Contributions

AA conceived the idea, revised the literature and drafted the manuscript. CM, FL-R, AZ, MG, and JH supervised the work and provided critical suggestions on the article. All authors read and approved the final version of the article.

## Conflict of Interest Statement

The authors declare that the research was conducted in the absence of any commercial or financial relationships that could be construed as a potential conflict of interest.
